# Social and psychological effects of a stoma on the sexuality and self-image of patients

**DOI:** 10.1007/s00384-026-05091-0

**Published:** 2026-01-20

**Authors:** Marvin Fischer, Jens Büntzel, Jutta Hübner

**Affiliations:** 1 Klinik Für Innere Medizin II, Hämatologie und Internistische Onkologie Universitätsklinikum Jena, Am Klinikum 1, 07747 Jena, Germany; 2 Südharz Klinikum Nordhausen gGmbH, Klinik Für Hals-Nasen-Ohrenheilkunde, Dr.-Robert-Koch-Straße 39, 99734 Nordhausen, Germany

**Keywords:** Stoma surgery, Quality of life, Sexual health, Body image, Psychosocial impact

## Abstract

**Purpose:**

Examining the psychosocial impact of stoma surgery on quality of life, sexual health, and body image, and analyzing demographic factors associated with these outcomes.

**Methods:**

This cross-sectional study included 214 adults with a stoma. Validated instruments, the European Organization for Research and Treatment of Cancer Quality of Life Questionnaire Core 30 (EORTC QLQ-C30), the Colorectal Cancer Module (EORTC QLQ-CR29), and the Sexual Health Module (EORTC QLQ-SH22), were used to assess quality of life, body image, and sexual function. Associations between demographic variables and patient-reported outcomes were tested using analysis of variance (ANOVA).

**Results:**

Participants reported considerable impairments with fatigue, emotional well-being, sexual interest, and body image. Global health status was significantly lower in women (*p* = 0.037); female participants also expressed significantly greater concerns regarding partner satisfaction (*p* = 0.027). Fatigue was significantly associated with age and gender (*p* = 0.032). Reduced sexual interest was reported by 36.9% of respondents, while 47.7% reported body image dissatisfaction.

**Conclusion:**

Stoma surgery is associated with substantial psychosocial and sexual health challenges, particularly in women and older adults. These findings underline the need for tailored postoperative support focusing on emotional well-being, intimacy, and body image.

## Introduction

Stoma formation is a common intervention in the management of colorectal cancer and chronic inflammatory bowel diseases. While the procedure is often essential for symptom control or oncological safety, individuals living with a stoma frequently experience substantial psychosocial challenges affecting emotional well-being, social participation, sexual functioning, and self-perception [1–9]. These issues can persist long after surgery and represent a major component of the patient’s overall quality of life [6–9].

Sexual dysfunction, reduced intimacy, and disturbed body image are recurrent problems reported by stoma patients in this study. Prior research indicates that these concerns may significantly affect self-esteem, relationship dynamics, and psychological health [3, 8–13]. Our research indicates that women appear to be particularly vulnerable, often describing greater distress related to physical appearance and partner acceptance. Age-related differences also contribute to variability in outcomes, with older adults more frequently reporting declines in sexual activity, lower resilience, and greater fatigue [14, 15]. Despite these documented problems, sexual health and self-image remain under-addressed in routine postoperative care, and communication between patients and healthcare professionals on these issues is often limited [5, 15].

Although quality of life research in stoma patients has grown, studies that simultaneously examine psychosocial well-being, body image, and sexual health across both cancer and non-cancer populations remain scarce. Research applying validated multidimensional instruments, such as the EORTC QLQ-C30, EORTC QLQ-CR29, and EORTC QLQ-SH22 [16–18], in Central European stoma populations is also severely limited. This creates a gap in understanding how demographic factors, including age, gender, and educational background, relate to postoperative psychosocial adaptation.

Our present study addresses this gap by assessing quality of life, sexual health, and body image in adults with a stoma using validated patient-reported outcome measures. We further analyzed demographic influences on these outcomes to identify groups experiencing heightened psychosocial burden. These findings aim to support the development of more targeted and patient-centered postoperative care strategies.

## Methods

This study was designed as a cross-sectional observational investigation to assess psychosocial and sexual health outcomes in adults with a permanent or temporary stoma.

Data collection took place between June 2022 and August 2024. The survey was administered digitally via SoSci Survey and hosted by the Clinic for Internal Medicine II of the University Hospital Jena. Although the study gave a paper-based alternative for accessibility purposes, all participants completed the questionnaire digitally. Participation was voluntary and anonymous; informed consent was obtained in the beginning, prior to accessing the survey.

Adults aged 18 years or older with a history of ileostomy or colostomy due to colorectal cancer, inflammatory bowel disease, or other diseases leading to an ileostomy or colostomy were eligible as participants. Participants were additionally required to have lived with a stoma for at least 6 months and to have sufficient cognitive ability to complete the questionnaire. Individuals younger than 18 years, those without prior stoma surgery, those with cognitive impairment preventing completion of the survey, and those who declined consent were excluded from the study. Recruitment occurred through German support groups for individuals with colostomies and ileostomies, allowing access to a broad and diverse patient population.

The primary outcomes of interest were quality of life, colorectal cancer–specific symptoms, body image, and sexual health. These were assessed using the EORTC QLQ-C30, EORTC QLQ-CR29, and EORTC QLQ-SH22, respectively. We additionally recorded demographic and clinical explanatory variables including age, gender, educational background, and underlying disease as well as the type of stoma, duration since surgery, and oncological treatment status.

All instruments used were official, German-validated versions of the respective EORTC questionnaires [16–18]. The EORTC QLQ-C30 assesses global health status, functional domains, and symptom burden [16]. The EORTC QLQ-CR29 evaluates gastrointestinal symptoms, stoma-related problems, and body image [17]. The EORTC QLQ-SH22 measures sexual activity, sexual satisfaction, intimacy concerns, and physical discomfort [18]. Scoring was performed in accordance with the EORTC scoring manual, with all scores transformed to standardized values ranging from 0 to 100.

Incomplete questionnaires were analyzed using available-case analysis. Missing values within multi-item scales were handled according to the EORTC scoring manual: scale scores were calculated when at least half of the items in a scale were completed, using the mean of available items.

Several steps were taken to reduce potential sources of bias. Selection bias was minimized by recruiting participants through multiple national patient support groups, allowing access to a broad spectrum of stoma patients. Information bias was addressed by using validated instruments (EORTC QLQ-C30, EORTC QLQ-CR29, EORTC QLQ-SH22) with standardized wording and scoring. Misclassification was unlikely, as all variables were collected via uniform digital input fields without interviewer involvement. To reduce analytical bias, assumptions for parametric testing were evaluated, including residual normality, variance homogeneity, and outlier inspection. No imputation methods were used to avoid artificial distortion of questionnaire scales.

The study size was determined by the number of eligible participants who completed the questionnaire during the recruitment period. No formal sample size calculation was conducted, as this was an exploratory cross-sectional survey aimed at describing psychosocial outcomes in a real-world patient population. Nevertheless, the final sample of over 200 participants exceeds the recommended minimum sample size for stable estimates in analyses using EORTC instruments [19].

Before conducting parametric analyses, statistical assumptions were evaluated. Residual normality was verified through visual inspection of standardized residual histograms, which showed an approximately normal distribution. Homogeneity of variances was assessed using Levene’s test. No extreme outliers were identified. Therefore, ANOVA was considered appropriate for group comparisons.

### Statistical analysis

Descriptive statistics, including means, standard deviations, frequencies, and percentages, were used to summarize the sample. Group comparisons of quality of life and sexual health outcomes were conducted using one-way analysis of variance (ANOVA) after assumptions for parametric testing were satisfied. A significance threshold of *p* ≤ 0.05 was applied. All analyses were conducted using IBM SPSS Statistics Version 29.0.2.0.

### Ethical considerations

This study was conducted in accordance with the Declaration of Helsinki. Ethical approval was obtained from the institutional review board (IRB) of the University Hospital Jena. Informed consent was given by all participants by answering the questionnaire. Participants were informed that the survey was anonymous.

## Results

### Demographic data

Of the 1473 patients reached by our survey, 214 filled out the questionnaires partially and 86 fully completed the questionnaires. 54,1% of patients were female, 43,6% of patients were male and 2,3% identified as diverse (Table 1). The median age was 44.6 years (range of 19 to 86). Participants had varying educational backgrounds, with the largest group holding a middle school diploma (34.1%). A considerable portion of participants had also completed higher education (22.7% with university degrees).

**Table 1:** Age distribution of participants, gender distribution of participants, and educational background of participants (*N* = 214)

Category	Group	Non cancer patients	Cancer patients	Number of responses	Percent
Age	18–30	18	9	27	12.6
	31–40	20	3	23	10.7
	41–50	25	8	33	15.4
	51–60	12	17	29	13.6
	61–70	3	9	12	5.5
	71–80	2	3	5	2.3
	81+	0	1	1	0.5
	Total	80	50	130	60.8
Gender	Female	53	19	72	33.6
	Male	25	31	58*	27.1
	Diverse	2	1	3	1.4
	Total	80	51	133*	62.2
Educational background	Secondary school certificate	10	6	16	7.5
	Secondary school leaving certificate	27	18	45	21.0
	A-levels or entrance qualification	24	13	38*	17.8
	Degree from university	17	13	30	14.0
	Other	2	1	3	1.4
	Total	80	51	132*	61.7

The majority of participants had either Crohn’s disease (31.6%) or colitis ulcerosa (15.4%). Of all participants, 16.2% were currently undergoing oncological treatment, while 24.3% had completed such treatments

*The discrepancy between the total values is attributable to incomplete data entries

### EORTC questionnaires

The analysis included summary measures for all EORTC outcome scales. Valid scores were available for 214 participants for the EORTC QLQ-C30 domains, 205 participants for the EORTC QLQ-CR29 scales, and 132 participants for the EORTC QLQ-SH22 sexual health scales. For each domain, mean values, standard deviations, and sample sizes were reported. As the study used questionnaire-based continuous scales, no outcome “events” occurred; instead, all results are presented as summary measures.

### EORTC QLQ-C30 quality of life outcomes

Overall, 50.9% of participants reported moderate to high fatigue, while 5.6% experienced severe fatigue. Pain was a concern for a subset of respondents, with 10.3% reporting severe pain, whereas 50.9% reported no or only minimal pain. Limitations in physical functioning were frequent: 63.4% reported difficulties with vigorous activities, and 19.8% experienced moderate difficulties with basic activities. Emotional distress was also present, with 20.5% reporting moderate to high emotional strain.

Age, gender, and education were entered simultaneously in the multifactorial ANOVA model for fatigue. Fatigue showed a significant correlation with all three factors (*F* = 3.570, *p* = 0.032). Older adults and women reported higher fatigue scores. Education showed a smaller, non-significant influence.

For physical functioning, the combined influence of age, gender, and education showed statistical significance (*F* = 2.695, *p* = 0.050). Individuals with higher educational levels tended to report significantly better physical functioning, although age and gender remained the more influential demographic factors.

Intimacy satisfaction showed a statistically significant association with gender (*F* = 3.962, *p* = 0.050), with women reporting lower satisfaction, frequently linked to body image concerns.

Global health status is significantly associated with gender (*F* = 4.455, *p* = 0.037), with women reporting lower global health scores. However, age and gender together (*p* = 0.110) and the combination of age, gender, and education (*p* = 0.156) did not show significant effects.

For fatigue, neither gender alone (*p* = 0.863) nor the combined effect of age, gender, and education (*p* = 0.071) showed a significant association.

### EORTC QLQ-CR29 colorectal-specific symptoms and body image

Bowel and urinary symptoms were reported frequently. Mild bloating occurred in 29.4% of respondents, and 17.6% reported severe bloating. Most participants (73.5%) experienced no urinary pain, while 3.1% reported frequent discomfort. Body image concerns were notable, with 47.7% expressing dissatisfaction with their physical appearance.

Sexual health was also affected: 36.9% of respondents reported reduced sexual satisfaction, and 14.3% reported a substantial decline in sexual interest.

Intimacy satisfaction showed a statistically significant association with gender (*F* = 3.962, *p* = 0.050), with women reporting lower satisfaction, frequently linked to body image concerns.

Intimacy satisfaction showed no significant effect of age and gender (*p* = 0.245).

Partner satisfaction concerns showed no significant effect of age and gender (*p* = 0.082), nor of the combined influence of age, gender, and education (*p* = 0.113).

### EORTC QLQ-SH22 sexual health outcomes

Reductions in sexual functioning were reported across several domains. Decreased sexual interest was noted by 36.9% of participants, while only 14.3% reported full satisfaction with their level of desire. Communication barriers were frequent: 56.3% of respondents had not discussed sexual health concerns with healthcare providers.

Physical discomfort during sexual activity was reported by 17.9% of participants; 27.4% expressed anxiety about potential discomfort. Among participants older than 60 years (*n* = 72), sexual activity had decreased in 54%.

## Discussion

The study aimed to examine how psychosocial, functional, and sexual health outcomes vary across demographic groups in individuals living with a stoma. Using the validated EORTC QLQ-C30, EORTC QLQ-CR29, and EORTC QLQ-SH22 instruments, which provide reliable and standardized measurement across multiple quality of life domains, the study yielded robust and clinically meaningful findings that align with our objectives. The results showed that several EORTC QLQ-C30 domains, particularly fatigue, physical functioning, and global health status, varied significantly by age and gender, with older adults and women reporting poorer outcomes. Educational level showed smaller yet significant associations in selected models.

Colorectal-specific symptoms and body image concerns (EORTC QLQ-CR29) were prevalent across the sample, yet only a limited number of these outcomes showed significant associations with demographic factors. Intimacy satisfaction and partner-related concerns demonstrated significant gender differences, with women reporting higher levels of dissatisfaction and insecurity.

Sexual health outcomes (EORTC QLQ-SH22) indicated reduced sexual interest, limited communication with healthcare professionals, and age-related reductions in sexual activity; however, most sexual health domains did not show statistically significant demographic effects.

Overall, our key findings indicate that demographic factors, particularly age and gender, play an important role in selected dimensions of quality of life and sexual well-being, whereas other symptom-related and psychosocial domains appear less severely influenced by these variables.

Several limitations should be considered when interpreting the findings of this cross-sectional study. First, due to the observational design, causal relationships cannot be inferred, and the identified associations between demographic factors and quality of life outcomes may be influenced by unmeasured variables. Second, data was collected through a self-administered online survey, which may introduce selection bias. Individuals with higher health literacy, digital access, or greater motivation to participate may be overrepresented. Information bias is also possible due to the reliance on self-reporting, although the use of validated EORTC instruments reduces this risk by providing standardized and reliable measurements.

Third, additional potentially relevant variables, such as comorbidities, socioeconomic status, psychological distress, stoma-related complications, or time since surgery, were not included in the analyses and may influence psychosocial and sexual health outcomes.

Despite these limitations, the use of validated EORTC questionnaires strengthens the reliability, reproducibility, and interpretability of the findings by ensuring that symptom burden, psychosocial functioning, and sexual health outcomes are measured consistently across participants.

The results of this study show that stoma surgery has substantial psychosocial consequences, particularly in domains related to global health, physical functioning, fatigue, emotional well-being, body image, intimacy, and sexual health. Consistent with our study objectives, several demographic factors were associated with specific quality of life outcomes. Gender emerged as an important determinant across multiple domains. Women reported significantly higher levels of emotional distress, more pronounced body image concerns, and greater dissatisfaction with intimacy and sexual life. These findings reflect gender-specific vulnerabilities and align with previous evidence highlighting that female stoma patients often experience stronger disturbances in self-image and intimacy after surgery [1–3, 7].

Fatigue and physical functioning were significantly associated with both age and gender, with older adults and women reporting higher fatigue and greater limitations in physical functioning. These patterns suggest that demographic characteristics shape the degree to which individuals experience functional and psychosocial consequences after stoma surgery. Educational level appeared to play a smaller, yet still significant role, although individuals with higher education tended to report somewhat better adaptation, which may reflect differences in health literacy or access to supportive resources.

Compared with earlier studies focusing mainly on global quality of life [2, 3, 11], the present findings extend the evidence by providing a more detailed assessment of sexual health and body image outcomes following stoma surgery. The more pronounced deficits observed in these domains underscore the importance of addressing sexuality and self-image as integral components of postoperative support. Differences between men and women were particularly evident in sexual self-perception and intimacy-related concerns, while concerns about partner satisfaction were present across genders, indicating the need for psychosocial and relational support interventions.

Age-specific patterns also emerged, with older adults showing significantly reduced sexual interest and activity consistent with age-related physiological changes and generational discomfort discussing sexuality [10, 15, 20], which are factors described previously in studies of stoma patients and older adult populations [14, 15]. The limited communication observed between patients and providers in this study reflects a persistent gap that remains widely reported in clinical practice [10, 12]. Given the high prevalence of sexual health difficulties in this population, improved patient to provider communication is essential for delivering appropriate education and support [21].

Overall, the study indicates that while many psychosocial challenges are widely shared among stoma patients, demographic characteristics, particularly age and gender, substantially shape both the severity and the clinical expression of these difficulties. These demographic disparities highlight critical areas of vulnerability and underscore the need for tailored, patient-centered interventions to address the unequal psychosocial burden experienced across different groups.

## Data Availability

Data is available from the first author on reasonable request.

## Appendix

Data is available from the first author on reasonable request.
